# The effect of prenatal counselling on postpartum family planning use among early postpartum women in Masindi and Kiryandongo districts, Uganda

**DOI:** 10.11604/pamj.2015.21.138.7026

**Published:** 2015-06-22

**Authors:** Richard Mangwi Ayiasi, Christine Muhumuza, Justine Bukenya, Christopher Garimoi Orach

**Affiliations:** 1 Makerere University College of Health Sciences, School of Public Health Department of Community Health and Behavioural Sciences, Uganda; 2 Makerere University College of Health Sciences, School of Public Health Department of Epidemiology and Biostatistics

**Keywords:** Postpartum, contraception, family planning, contraceptive counselling

## Abstract

**Introduction:**

Globally, most postpartum pregnancies are unplanned, mainly as a result of low level of knowledge and fear of contraceptive use especially in low-income settings. The aim of this study was to evaluate the effect of prenatal contraceptive counselling on postpartum contraceptive use and pregnancy outcomes after one year.

**Methods:**

Sixteen health centres were equally and randomly allocated to control and intervention arms. Mothers were consecutively recruited during their first antenatal clinic consultations. In the intervention arm Village Health Team members made home visits and provided prenatal contraceptive advice and made telephone consultations with health workers for advice while in the control arm mothers received routine antenatal care offered in the health centres. Data were collected in 2014 in the two districts of Kiryandongo and Masindi. This data was collected 12-14 months postpartum. Mothers were asked about their family planning intentions, contraceptive use and screened for pregnancy using human Chorionic Gonadotropin (hCG) levels. Socio-demographic and obstetric indices were recorded. Our primary outcomes of interests were current use of modern contraceptive, decision to use a modern contraceptive method and pregnancy status. Multilevel analysis using the xtmelogit stata command was used to determine differences between intervention and control groups.

**Results:**

A total of 1,385 women, 748 (control) and 627 (intervention) were recruited. About 80% initiated breastfeeding within six hours of delivery 78.4% (control) and 80.4% (intervention). About half of the mothers in each arm had considered to delay the next pregnancy 47.1% (control) and 49% (intervention). Of these 71.4% in the control and 87% in the intervention had considered to use a modern contraceptive method, only 28.2% of the control and 31.6% in the intervention were current modern contraceptive users signifying unmet contraceptive needs among immediate postpartum mothers. Regarding pregnancy, 3.3% and 5.7% of the women were found to be pregnant in the control and intervention arms respectively. There were no statistical differences between the control and intervention arms for all primary outcomes of interests.

**Conclusion:**

Prenatal contraceptive counseling did not affect postpartum contraceptive use among immediate postpartum mothers in Masindi and Kiryandongo districts. Interventions aiming at improving postpartum contraceptive use should focus on addressing unmet contraceptive needs.

## Introduction

Pregnancy and childbirth are important stages in life because they are associated with social problems, mortality and morbidity of mother and the child especially in low income countries [1]. Inter-pregnancy interval under one year is a risk factor for preterm delivery and neonatal death [2]. However, in low-income settings unintended pregnancies commonly occur during the first twelve months postpartum [3]. Demographic and Health Surveys from 17 countries showed that up to 88% of the women in the first year postpartum would like to avoid pregnancy but are not accessing contraception [4]. The World Health Organization (WHO) recommends an interval of 24 months or more before attempting a next pregnancy after a live birth. This is meant to reduce adverse outcomes for the mother and the child [5]. But most women, especially in the postpartum period, have little or no understanding of fertility. Moreover, it is difficult to predict when an individual woman will become fertile again in the postpartum period. The most important factor influencing postpartum fertility is breastfeeding [6, 7]. Irregular breastfeeding and introduction of complementary feeding before six months compromises the effectiveness of breastfeeding as a natural method of contraception [4]. However, in situations where most women do not attend antenatal clinics and secure home deliveries the risk of postpartum pregnancy is largely unknown [8]. In Uganda, contraceptive policies are liberal and the total fertility rate remains high at 6.2. Only 26% of women use modern contraceptives and 61% have no access [9], while the rate of unwanted pregnancies especially among the married women is reported to be 41% [10]. Contraceptive use remains low for various reasons. For example a study conducted among prenatal women at the national referral hospital Mulago showed that women shared the incorrect notion that contraceptives can cause cancers and infertility [11]. The authors in this study suggest effective educational interventions to dispel the pervasive myths among poor communities. Deliberate promotion of postpartum family planning has shown promising results. Most of the evidence is limited to middle- and high-income countries [12]. In Mexico, for example, integration of family planning into prenatal services showed increased contraceptive use where the demand is high [13]. A systematic review recently published also showed that prenatal care, home visitation and educational interventions were associated with improved family planning outcomes [14]. Therefore programs promoting contraception counselling during pre- and postnatal care are important in improving family planning outcomes and preventing induced abortions [15, 16]. However this study suggests further exploration of the evidence in low resource settings [14]. We conducted a community intervention study in which 16 health centres were randomly allocated to intervention (8) and control (8) arms. Health centres were considered eligible for randomisation when they offered antenatal and delivery services. In the intervention arm we engaged community health workers (CHWs) In Uganda Community Health Workers are given the collective name of Village Health Teams-VHTs. Specifically for family planning, VHTs provided counselling on the risk of pregnancy soon after delivery; available options for delaying the next pregnancy and emphasised the importance of regular and exclusive breastfeeding as a means to delay pregnancy. The control arm received standard care that was routinely offered at the health centres. Routine care included group health education on general maternal and newborn issues offered during antenatal care visits [17]. The aim of this study was to determine the effect of prenatal family planning counselling on postpartum contraceptive use. Specifically, we assessed the decision to use modern contraceptive methods, the extent of contraceptive use and prevalence of pregnancy among postpartum women in Masindi and Kiryandongo districts in Western Uganda. We examined the extended postpartum period and limited ourselves to the first 12 months because of logistical limitations. The World Health Organisation considers the “ideal” postpartum period to be 24-47 months.

## Methods

Study design-this was a community intervention study.

### Study design and randomisation

In this study, sixteen health centres were randomly and equally allocated to control or intervention arms. Health centres were selected on the basis of offering maternal and newborn services. Names of relevant health centres were written on pieces of papers and rolled into a ball. Two people not related to the study assigned to represent control and intervention group were asked to randomly pick eight papers from the pool of 16. Blinding was not necessary since randomisation was at the level of health centres. Given the distribution of health centres in the two districts we could not guarantee absence of contamination.

### Study area and population

The study was conducted in Masindi and Kiryandongo districts in western Uganda. This study site was chosen because it is a study site for the first author (RMA) who is a doctoral student conducting his studies in the same area. The region is located 214 kilometres from the capital Kampala. The population is about 700,000 inhabitants with 96% of them living in the rural parts of the region. There are 42 health centres; two district hospitals, one health centre IV, 16 health centres IIIs and the rest were health centres II. Previous studies conducted in this region showed that ANC first attendance was nearly universal with 97% making at least one ANC visit during their pregnancy, but less than 50% of them deliver with the help of a skilled attendant [18]. This was part of a larger study that was evaluating maternal and newborn effects of home visits combined with mobile phone consultations carried out by VHTs.

### Selection of study participants

Study participants were pregnant women who made antenatal visits to the health centre at a gestational age of 28 weeks or less. Gestational age was estimated by palpating the height of fundus. All pregnant women that qualified were eligible for enrolment. We did not devise exclusion criteria.

### Data collection

Fifteen research assistants were recruited and trained for two days on the tools and on techniques of data collection. Research assistants visited the VHTs or local counsellors in the village for direction. In the control arm the search for recruited women was not easy because there was modest prior interaction between VHTs and respondents. Consequently physical addresses could not be easily located. For the intervention women, VHTs led research assistants to their respective homes because they had already interacted with most of the women in the area.

### Data analysis

Data was entered in data entry screen in epidata and later transferred to stata for data cleaning and further analysis. First the baseline characteristics of the control and intervention arms were compared using two-by-two tables. Outcome measures of interest were willingness to delay subsequent pregnancy, decision on what contraceptive methods to use, postnatal contraceptive use and postpartum pregnancy within one year.

### Ethical considerations

A written consent was secured from all the women that were recruited in the control and intervention arms of the study. When pregnant women arrived at the health centre they were screened and those found to be eligible were requested to participate in the study. This consent process was launched by the midwife or nurse on duty. After consenting a personal file for the woman was opened at the health centre. After enrolling women in the intervention group, the midwife called the VHT alerting him/her about the presence of a pregnant woman who has been recruited. During data collection, the women were reminded about their prior consent and a verbal consent was obtained to volunteer information for the second phase of data collection. This study was approved by the higher degrees and research committee of the school of public health and the national council of science and technology, Kampala Uganda. The study was registered with clinicaltrials.gov as NCT02084680.

## Results

In the intervention arm 402/627 (64.1%) women reported having received at least one prenatal visit and 328/627 (54.6%) reported postnatal visit by the VHT; 156/627 (24.9%) reported newborn illness. Among the control group 138/ 758 (18.2%) and 147/758 (26.0%) reported at least one VHT prenatal and postnatal visits respectively; 192/758 (25.3%) reported newborn illness within the first 28 days of life.

### Baseline characteristics

Baseline characteristics such as age, level of education, religion, marital status and other past obstetric characteristics were collected. At baseline, some household characteristics were comparable (Table 1). For example, 90.1% vs. 92.8% were Christians; attained at least secondary education level 16.2% vs. 16.3%; were living with spouses 90.8% vs. 86% in the control and intervention groups respectively. In the obstetric characteristics those that attended at least one antenatal visit 95.9% vs. 94.7%; had a term normal delivery 93% vs. 94.1% and gestation age at first antenatal visit within the first trimester 91.3% vs. 88.8% among control and intervention groups.

**Table 1:** Percent distribution of study participants by background characteristics

	Control n (%)	Intervention n (%)	*p*-value
**Household characteristics n = 1,385**			
**Age of mother**			
13-24 yrs	413 (54.5)	377 (60.1)	
> = 25 yrs	345 (45.5)	250 (39.9)	0.035
**Ethnicity**			
Migrant	593 (78.2)	424 (67.6)	
Indigenous	165 (21.8)	203 (32.4)	<0.001
**Religion**			
Other religion	75 (9.9)	45 (7.2)	
Christian	683 (90.1)	582 (92.8)	0.170
**Education**			
None/primary	635 (83.8)	525 (83.7)	
Secondary/tertiary	123 (16.2)	102 (16.3)	0.984
**Marital status**			
Single/separated	70 (9.2)	88 (14.0)	
Living with spouse	688 (90.8)	539 (86.0)	0.005
**Source of income**			
None/housewife	365 (48.2)	359 (57.3)	
Regular/stable income	393 (51.8)	268 (42.7)	0.001
**Selected Obstetric characteristics**			
**Number of pregnancy**			
Second/more	552 (72.8)	363 (57.9)	
First	206 (27.2)	264 (42.1)	<0.001
**Number of ANC visits in previous pregnancy (n = 1,085)**			
0-3 visits	238 (37.8)	231 (50.7)	
4/more visits	391 (62.2)	225 (49.3)	<0.001
**Outcome of last pregnancy**			
Abortion/stillbirth	44 (7.0)	27 (5.9)	
Term/live baby	585 (93.0)	429 (98.1)	0.480
**Where last delivery took place (n = 1,046)**			
Home/way to facility	314 (52.1)	204 (46.1)	
Health facility	289 (47.9)	239 (53.9)	0.054
**Attendant at birth**			
None professional	301 (49.9)	184 (41.5)	
Professional health worker	302 (50.1)	259 (58.5)	0.007
**Gestation age at recruitment**			
> 20 weeks	381 (50.3)	346 (55.2)	
≤ 20 weeks	377 (49.7)	281 (44.8)	0.068
**Level of health centre**			
Level II	514 (67.8)	198 (31.6)	
Level III	244 (32.2)	429 (68.4)	<0.001

### Postpartum practices and contraceptive use and pregnancy status

There was no difference in breastfeeding practices between control and intervention groups (see Table 2). Two fifths of the mothers, 78.4.6% and 80.4% among control and intervention mothers initiated breastfeeding within six hours after giving birth. The relative risk of initiating breastfeeding within six hours was slightly higher among the intervention group but this difference was not statistically significant (aRR; 1.02: 95% CI: (0.97-1.08); p-value = 0.376). Nearly three-quarters of the women in each arm, 74.5% among control and 73.7% among intervention, avoided pre-lacteal feeds soon after giving birth. There was no statistical difference in the practice of offering pre-lacteal feeds (aRR: 0.99: 95%CI: (0.93-1.05) p-value 0.745). About half of postpartum women, 47.1% (control) and 49% (intervention) arm had considered delaying the next pregnancy among the current none contraceptive users signifying unmet needs for contraceptive use. Of these 71.4% among control and 87% in the intervention had considered using a modern method of contraceptive. In the preliminary analysis, the risk of being willing to use was one and half times higher among the intervention group, but this difference was not statistically significant after adjustment (aRR: 0.98; 95%CI: (0.53-1.82); p= 0.955). Only 28.2% (control) and 31.6% (intervention) of mothers were current users of modern contraceptives. Although there was slightly higher proportion of current users in the intervention arm, this difference was not statistically significant (aRR: 1.10; 95%CI: (0.51-1.82); p= 0.810). When we examined individual methods of family planning and compared between control and intervention arm, the greatest difference was seen in lactation amenorrhoea whereby intervention mothers were 16 times more likely to report lactation amenorrhoea compared with control mothers p<

**Table 2:** Distribution of women by breastfeeding practices, contraceptive use and pregnancy status

Indicator	Control	Intervention	uRR (95% CI)	p-value	aRR (95%CI)	p-value
**Breastfeeding practices**						
**Initiation of breastfeeding**						
After 7 hrs./more	160 (21.6)	118 (19.6)				
Within 6 hrs.	581 (78.4)	483 (80.4)	1.02[0.97-1.08]	0.376	1.16(0.51-2.61)	0.719
**Offer of pre-lacteal feeds**						
Gave pre-lacteal feeds	189 (25.5)	158 (26.3)				
Did not give pre-lacteal feeds	552 (74.5)	443 (73.7)	0.99[0.93-1.05]	0.745	1.09(0.56-2.03)	0.802
**Contraceptive practices**						
**Current users**						
Traditional methods	526 (71.8)	397 (68.5)				
Modern methods	207 (28.2)	183 (31.6)	1.04 (0.40-2.70)	0.942	1.10(0.51-2.40)	0.810
**Willing to use**						
Not considered	263 (52.9)	152 (51 .0)				
Yes	234 (47.1)	146 (49.0)	0.94 (0.52-1.71)	0.842	0.98(0.53-1.82)	0.955
**Willing with a chosen method**						
Traditional methods	67 (28.6)	19 (13.0)				
Modern contraceptive	167 (71.4)	127 (87.0)	1.50 (0.41-5.66)	0.522	0.91(0.18-4.67)	0.914
**Pregnancy test**						
Positive test	25 (3.3)	36 (5.7)				
Negative test	733 (96.7)	591 (94.3)	0.52 (0.14-1.90)	0.324	0.50(0.13-1.87)	0.302

## Discussion

Our results show that there was no difference in the control and intervention arms regarding contraceptive use among postpartum women. However, a high proportion of women had made a decision to use modern contraceptive methods although they were not yet using it. This may be due to a number of reasons. First, there could have been limited supply of essential medicines at the health facility including contraceptives. Moreover, VHTs were not provided with contraceptive supplies to offer to the women. Current users could have been higher if VHTs counselled mothers and simultaneously offered alternative contraceptive methods. This observation could be highlighting the problem of unmet family planning needs among postpartum mothers in Masindi and Kiryandongo districts. Second, mothers could be facing the mixed feelings about side-effects and myths surrounding contraceptive use despite the training by VHTs. This is in line with results from a review of Demographic and Health Surveys from 17 countries that indicate that nearly two-thirds of women in their first postpartum year have an unmet need for family planning. The review also indicates that return to sexual activity is associated with the return of menses, breastfeeding status, and postpartum duration but not generally associated with contraceptive use [19]. We were surprised that prenatal home visits and counselling on postpartum contraception did not affect utilisation of modern contraceptives demonstrated by non-significant statistical tests between the control and intervention arms. However, similar results were reported in Turkey [20] although this was a different context from ours. Similarly, in a multi-centre study involving Edinburgh Scotland, Cape Town south Africa and Shanghai China the authors could not demonstrate any difference in prevalence of contraceptive use and postpartum pregnancy [21].

A systematic review showed that there were differences between control and intervention groups regarding the use of different methods of modern contraceptives. For example, in some instances intervention groups were less likely to use injectable contraceptives compared with the control group [22]. Our analysis could not show significant results partly because we categorised all modern contraceptives as one instead of discerning the different contraceptive methods in which there could be variations in choices between control and intervention women. Also, our intervention provided counselling on contraceptives without offering Family Planning services or referrals for contraceptives and this could have limited actual use. Frustration for low access to family planning methods among intervention mothers could partly explain the higher proportion of pregnancy in the early postpartum period among intervention mothers. However, when we disaggregated individual family planning methods mothers reporting lactation amenorrhoea were significantly higher in the intervention compared with control mothers. This was expected because VHT education emphasised early initiation and exclusive breastfeeding. It was encouraging to note that only 13% of the mothers in the intervention arm were planning to use traditional methods of contraception compared with 28% in the control arm. Traditional methods such as lactation amenorrhoea are considered to be effective when three conditions are simultaneously present, the baby is less than six months old, exclusive breastfeeding on demand and at least five times a day and the mother is amenorrhoeic. This can only be achieved in highly motivated individuals or couples. Non-significant differences between treatment arms could be a result of the low level of VHT training, skills and capability to effectively explain technical services like family planning as part of their expected duties. Also VHTs in the control group were providing home visits and counselling as their routine duties, they may have offered counselling on family planning as part of their expected duties. This may have contributed to the lack of significant behaviour difference between treatment arms. Among the category “current users” women who reported they were not using modern contraceptive methods were grouped under the option “traditional methods” and this could have affected the level of significance of this test.

## Conclusion

The aim of this study was to evaluate the effect of prenatal family planning counselling on postpartum family planning use among early postpartum mothers. Analysis did not show significant differences between control and intervention arms in the decision to use family planning, family planning choices and actual use of family planning methods. Our results contribute to the existing evidence that family planning counselling per se may not influence the prevalence of modern contraceptive use. Interventions aiming to promote postpartum family planning use should simultaneously target increasing access to information and methods because there is a large proportion of mothers who have expressed willingness to use and have made the decision on what methods to use but are not accessing the methods.

**Strength and limitations:** This study is important because it addressed the problem of postpartum contraception antenatally. Most women encounter a brief postpartum contraceptive counselling soon after delivery at a time when they are preparing to go home and this is considered to be less effective.
